# Fatal liver mass rupture in a common-variable-immunodeficiency patient with probable nodular regenerating hyperplasia

**DOI:** 10.1186/s13223-021-00643-1

**Published:** 2022-01-07

**Authors:** Mongkhon Sompornrattanaphan, Ranista Tongdee, Chamard Wongsa, Anupop Jitmuang, Torpong Thongngarm

**Affiliations:** 1 Division of Allergy and Clinical Immunology, Department of Medicine, Faculty of Medicine Siriraj Hospital, Mahidol University, 2 Wanglang Road, Bangkok Noi, Bangkok, 10700 Thailand; 2 Department of Diagnostic Radiology, Faculty of Medicine Siriraj Hospital, Mahidol University, Bangkok, Thailand; 3 Division of Infectious Diseases and Tropical Medicine, Department of Medicine, Faculty of Medicine Siriraj Hospital, Mahidol University, Bangkok, Thailand

**Keywords:** Common variable immunodeficiency, Hepatic rupture, Immunodeficiency, Nodular regenerating hyperplasia, TACI mutation

## Abstract

**Background:**

Nodular regenerating hyperplasia (NRH) is the most common liver involvement in common variable immunodeficiency (CVID**).** Most patients are asymptomatic with gradually increasing alkaline phosphatase (ALP) and mildly elevated transaminase enzymes over the years. We report the first case of fatal liver mass rupture in a CVID patient with probable NRH**.**

**Case presentation:**

A 24-year-old man was diagnosed with CVID at the age of 1.25 years. Genetic testing revealed a transmembrane activator and calcium-modulator and cyclophilin-ligand interactor (TACI) mutation. He had been receiving intravenous immunoglobulin (IVIg) replacement therapy ever since then. The trough level of serum IgG ranged between 750–1200 mg/dL. However, he still had occasional episodes of lower respiratory tract infection until bronchiectasis developed. At 22 years old, computed tomography (CT) chest and abdomen as an investigation for lung infection revealed incidental findings of numerous nodular arterial-enhancing lesions in the liver and mild splenomegaly suggestive of NRH with portal hypertension. Seven months later, he developed sudden hypotension and tense bloody ascites. Emergency CT angiography of the abdomen showed NRH with intrahepatic hemorrhage and hemoperitoneum. Despite successful gel foam embolization, the patient died from prolonged shock and multiple organ failure.

**Conclusions:**

Although CVID patients with NRH are generally asymptomatic, late complications including portal hypertension, hepatic failure, and hepatic rupture could occur. Therefore, an evaluation of liver function should be included in the regular follow-up of CVID patients.

## Background

Common variable immunodeficiency (CVID) is characterized by impaired B-cell differentiation, causing decreased plasma cells and low levels of immunoglobulin production while circulating absolute B cell counts and T cell functions are usually normal [1]. Clinical features of CVID are highly heterogeneous, including infections primarily caused by bacteria in the respiratory and gastrointestinal tracts, autoimmune disorders despite the decreased antibody production, and malignancies. Immunoglobulin replacement therapy protects most patients against infection but does not protect them against non-infectious complications in which their prevalence increases as CVID patients live longer [2].

The prevalence of non-infectious liver diseases in a large CVID cohort in the United States (US) was 12.7%, and granulomas and nodular regenerative hyperplasia (NRH) were the most common pathological features [3]. The prevalence of abnormal liver function with raised alkaline phosphatase (ALP) levels in a CVID cohort in the United Kingdom (UK) was 43.5%, mostly due to NRH [4]. Of interest, liver disease in CVID patients was associated with increased mortality [3]. NRH is common in CVID patients, yet fatal hepatic rupture due to NRH has never been reported. Herein, we report a 24-year-old male CVID patient with probable NRH who died due to hepatic rupture.

## Case presentation

A 24-year-old Thai male initially presented with recurrent sinopulmonary tract infection and chronic diarrhea since the age of 11 months old. Investigation of his immune status revealed an IgG level of 330 mg/dL (normal range, 344–1180 mg/dL) while absolute B cell (CD19) count was 1883 cells/mm^3^ (normal range, 171–465 cells/mm^3^). His IgM and IgA were undetectable. The absolute CD4 and CD8 counts were 3050 cells/mm^3^ (normal range, 1000–4600 mm^3^) and 2773 cells/mm^3^ (normal range, 400–2100 mm^3^), respectively. Genetic testing revealed a transmembrane activator and calcium-modulator and cyclophilin-ligand interactor (TACI) mutation.

The diagnosis of CVID was established at the age of 1.25 years. He had been receiving intravenous immunoglobulin (IVIg) replacement therapy ever since then. The trough level of serum IgG ranged between 750 and 1200 mg/dL. However, he had occasional lower respiratory tract infection episodes until he developed bronchiectasis at 15 years old.

**Fig. 1:**
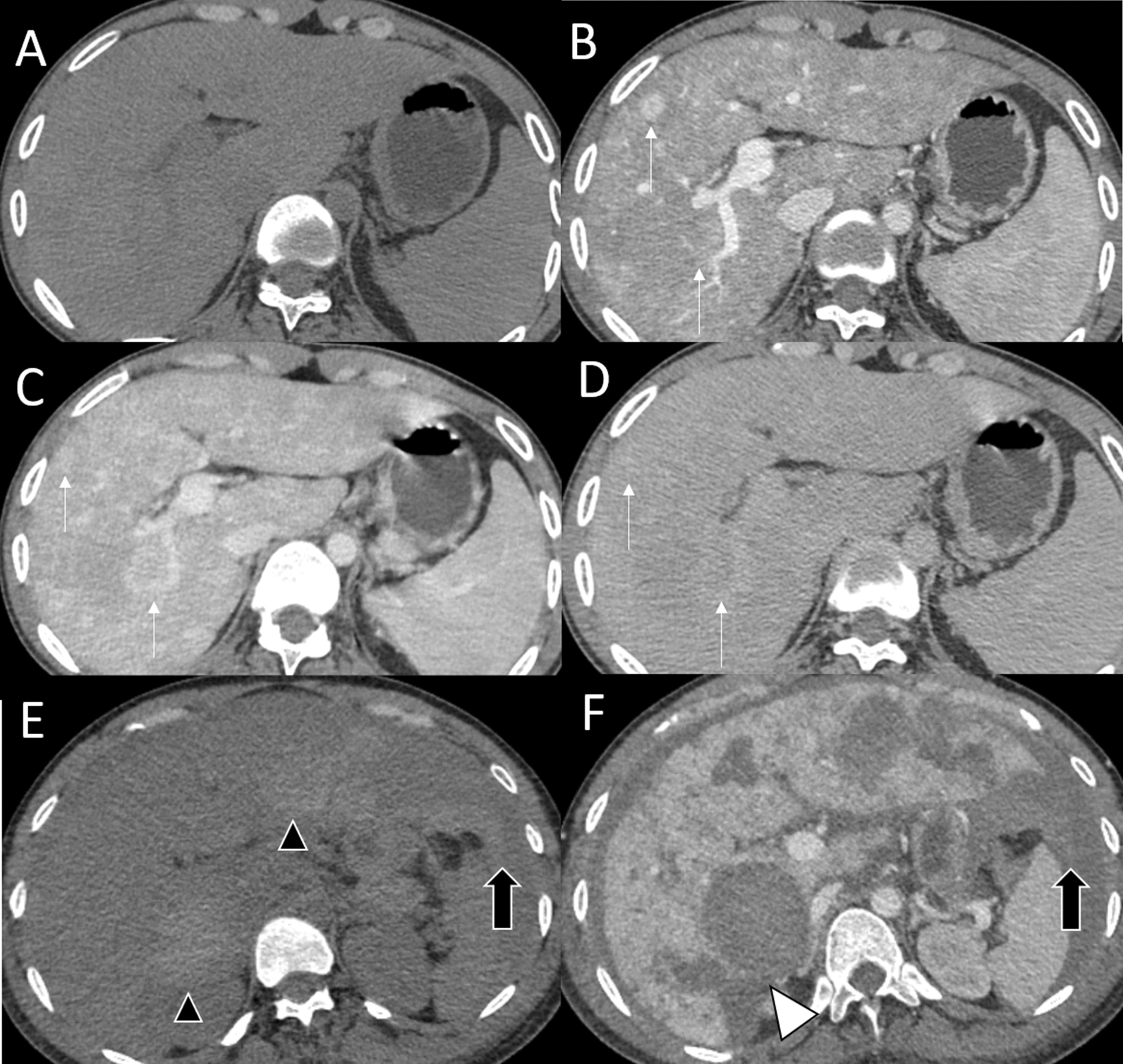
Hepatic imaging **A**–**D**. The initial multiphasic CT. **A** The non-contrasted image shows no abnormal intrahepatic bleeding. **B** The arterial phase shows multiple poorly-defined, arterial-enhancing nodules scattered throughout both hepatic lobes (white arrows). In portovenous phase **C** and 5 min-delayed phase **D**, these nodules remain iso- to hyperattenuating compared to the adjacent liver parenchyma. Hepatic imaging **E**–**F**. The 7-month follow-up CT. **E** The non-contrasted image shows multiple areas of intrahepatic bleeding (black arrowheads) and intraperitoneal bleeding (black arrows). **F** The portovenous phase demonstrates disruption of the liver capsule (white arrowhead), suggesting rupture of these hypervascular liver lesions

At 22 years old, he developed an intermittent and remittent fever for 1 month. No causative organism was identified. Diagnostic workup including paranasal sinus films and nasal endoscopy were negative for sinusitis. Computed tomography (CT) chest and abdomen suggested an active infectious process. Sputum AFB and sputum PCR for *Mycobacterium tuberculosis* (PCR-TB) were negative. However, tuberculosis is common in Thailand, so empirical treatment with anti-tuberculous (Anti-TB) drugs was prescribed. Two months later, a repeat CT chest suggested improved pulmonary pathology. However, incidental findings of numerous nodular arterial-enhancing lesions in the liver were demonstrated. Multiphasic CT liver imaging and mild splenomegaly suggested NRH with early portal hypertension (Fig. 1A–D). He was non-alcoholic and did not take any non-prescribed drugs. Viral hepatitis profiles (HBsAg, Anti-HCV IgM and IgG) were all negative. Seven months later, he was hospitalized with pneumonia. His IgG level was 890 mg/dL. On day 16 of the admission, he developed sudden hypotension. Physical examination revealed marked anemia and tense abdominal distension. Ultrasound-guided abdominal paracentesis yielded bloody fluid. Emergency CT angiography of the abdomen showed NRH with internal hemorrhaging and intramural pseudoaneurysm (Fig. 1E, F). Newly detected multiple areas of intrahepatic hemorrhage with rupture of the overlying Glisson’s capsule caused a large amount of hemoperitoneum. Although emergency angiography with gel foam embolization at the right and left hepatic artery was successful, the patient died from hemorrhagic shock and multiple organ failure.

**Fig. 2:**
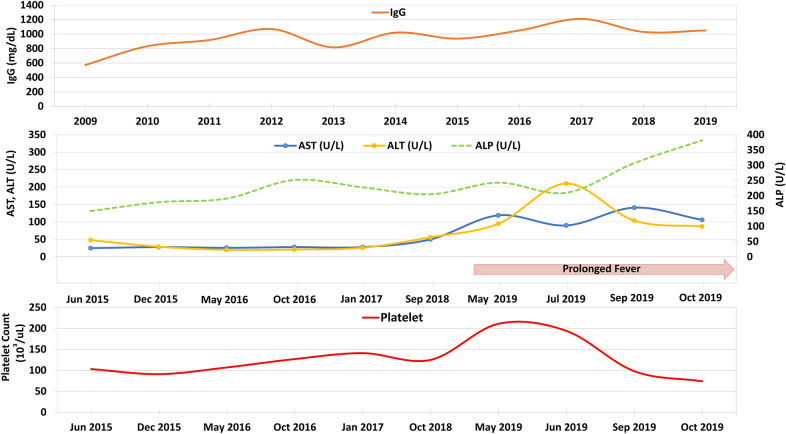
The trend of trough level of immunoglobulin G, liver enzymes, and platelet count during follow-up

The trends of serum IgG, aspartate transaminase, alanine transaminase, ALP, and platelet count are summarized in Fig. 2.

## Discussion and conclusions

We report a CVID patient experiencing a fatal hepatic rupture. Given the long period of ALP elevation and the CT imaging findings, NRH was the most likely diagnosis. Rupture is an uncommon complication of NRH. To our knowledge, this is the first case to be reported as a complication of NRH in CVID patients.

Liver impairment was present in 11.9% of CVID patients in a US cohort [5]. Seventy-nine percent of CVID patients in a UK cohort had abnormal laboratories, imaging, and/or histopathology of liver disease [6]. NRH is generally considered the most common liver involvement in CVID with a prevalence of 5–12% [4, 7]. It is thought to be caused by intra-hepatic vasculopathy, leading to hepatocyte injury and regeneration of characteristic nodules [8]. Abundant regenerative nodules can compress hepatic sinusoids, causing noncirrhotic portal hypertension [7]. Most patients are asymptomatic with gradually increasing ALP levels over the years [4]. Mildly elevated transaminases were reported in approximately 50% of patients [7]. Our patient’s laboratory results revealed a gradual increased ALP for several years, increased transaminases for 5 months, and a progressive platelet decline for 2 months before the event. The anti-TB drugs could result in drug-induced liver injury (DILI) in our patient. However, the timeline showed that the abnormal liver enzymes occurred before the initiation of anti-TB drugs but became slightly worsening 2 months after starting anti-TB drugs. The worsening liver enzymes could be due to multiple factors, including liver involvement of TB itself, pre-existing liver lesions, or DILI. However, anti-TB drugs could not solely explain the abnormal liver findings from CT scans.

Late complications include ascites, esophageal varices, and splenomegaly due to portal hypertension, usually appearing years after the elevated liver enzyme [4, 7]. CT features of NRH are multiple, arterial-enhancing liver nodules or masses typically becoming iso- or slightly hyperintense in the portovenous and delayed phases without rapid washout [9]. The histopathological feature of NRH is characterized by intrasinusoidal inflammatory cell infiltrates with CD3, CD4, CD8 T cells and portal vein endotheliitis, suggesting a possible role of T cells in the pathogenesis [7, 10]. Chronic CD8 cell infiltration would partially account for increased IFNγ production positively correlated with disease severity [7]. CVID and other primary humoral immunodeficiency are often associated with autoimmune diseases and lymphocyte abnormalities [10]. These findings combined with histopathologic features of intrasinusoidal T cell infiltrates suggest an autoimmune mechanism. The development of NRH in CVID patients is not associated with age at onset, age at diagnosis, and duration of immunoglobulin replacement therapy [4].

The differential diagnosis for multiple hypervascular hepatic lesions includes malignant hypervascular tumors, e.g., hepatocellular carcinoma (HCC) and benign lesions, e.g., hepatic adenoma (HA), hemangioma, and NRH. Although HCC is the most common hypervascular liver tumor causing spontaneous hepatic rupture, the absence of typical CT findings for HCC, including early enhancement with rapid washout in portovenous or delayed phase, makes the diagnosis unlikely in the present case. HA is commonly seen in young women and is associated with oral contraceptives or anabolic steroid use, making HA an unlikely diagnosis in the present case although the CT findings may support this diagnosis. Hemangioma typically shows peripheral nodular enhancement in the arterial phase and progressive contrast fill-in in the delayed phase, and none of those findings was seen in the present case. Given a gradual increase of ALP over time in this CVID patient with supportive CT imaging, the most likely diagnosis of NRH was reasonably made. The autopsy request, unfortunately, was declined due to the patient’s cultural issue. Taking the lesson learned from this case to clinical practice, patients with CVID or other humoral immunodeficiencies with either one or in a combination of these following features including (1) hepatomegaly; (2) splenomegaly; (3) persistently elevated liver enzymes; and (4) thrombocytopenia should undergo liver CT scans and investigate for other causes of liver injuries such as viral hepatitis and drugs. If the diagnosis of NRH is suspicious, liver specialist consultation to carefully consider liver biopsy should be performed. Prophylactic intervention might be considered in cases at a high risk of NRH rupture (e.g., a huge mass, mass located adjacent to the liver capsule, or history of previous rupture). The data on the risk–benefit ratio of prophylactic embolization in such cases was limited. Therefore, the decision should base on a case-by-case basis and supervised by the liver specialists. Our recommendation was concordant with the recent report by Nunes-Santos, et al. [11].

In conclusion, NRH is the most common liver involvement in CVID patients. Most CVID patients with NRH are asymptomatic with gradually increasing ALP levels, and NRH could lead to portal hypertension and a fatal rupture complication, as in the present case. Therefore, clinical examination of hepatosplenomegaly and evaluation of CBC and liver function should be included in the follow-up of CVID patients.

## Data Availability

Not applicable.

## Abbreviations

ALP: Alkaline phosphatase
AST: Aspartate transaminase
ALT: Alanine transaminase
IgG: Immunoglobulin G
